# In ‘hot’ pursuit: exploring the evolutionary ecology of labial pits in boas and pythons

**DOI:** 10.1098/rspb.2025.0199

**Published:** 2025-04-23

**Authors:** Aritra Biswas, Avrajjal Ghosh, Madhura Agashe

**Affiliations:** ^1^ Centre for Ecological Sciences, Indian Institute of Science, Bengaluru, Karnataka, India; ^2^ School of Biological Sciences, National Institute of Science Education and Research, Bhubaneswar, Orissa, India

**Keywords:** infrared sensing, key innovation, pit organ, macroevolution, python, boas

## Abstract

The evolution of thermoreception in animals, particularly that of infrared (IR)-sensing pits in boas, pythons and pit vipers, is a fascinating area of sensory ecology. While numerous studies have focused on the molecular mechanisms of IR sensing in snakes, the broader ecological and evolutionary significance remains less explored. In this study, we examined the origins and evolutionary consequences of labial pits in boas and pythons using phylogenetic comparative methods. We analysed how various ecological and biological factors—such as hunting mode, diet, habitat, body size and biome—were correlated with the presence of pits, and whether this adaptation influenced diversification rates. Our findings revealed that labial pits evolved multiple times and showed strong associations with an arboreal habitat and endothermic diet, but we did not find a significant correlation between pits and hunting mode or any other ecological traits. Moreover, lineages with pits did not exhibit higher diversification rates. This research provides new insights into the eco-evolutionary role of heat-sensing pits, suggesting that the emergence of labial pits might have acted as a key innovation, significantly affecting the evolution of habitat use patterns and prey preference for pythons and boas.

## 1. Introduction

The evolution of unique morphological and physiological traits can act as key innovations that provide access to new niches and competitive advantages to organisms [1]. These traits may be especially beneficial in stable environments, leading to increased diversification rates [2]. For instance, adhesive toepads are linked to arboreal specialization in lizards [3]. However, such traits can be disadvantageous during sudden climatic shifts due to genetic constraints, leading to extinction or evolutionary dead ends [4,5], as seen in the evolution of fossoriality in snakes [6]. Regardless of their impact, key innovations undeniably shape the evolutionary dynamics and trajectories of organisms [7].

One such notable innovation is the use of thermoreceptors in various animal groups such as pyrophilous beetles, certain butterflies, vampire bats and mosquitoes ([8,9] and references therein). Thermal receptors are specialized sensors that detect heat from the environment in the form of infrared (IR) radiation—electromagnetic energy with wavelengths longer than visible light but shorter than radio waves [10]. These receptors allow animals to perceive and respond to shifts in thermal energy, aiding in tasks such as finding prey, maintaining body temperature and avoiding heat damage [8,11].

Infrared-sensitive pits in snakes are among the most well-known examples of thermoreceptors. The connection between pits and IR radiation was first proposed by Ros in 1935, who conducted behavioural experiments on her pet *Python sebae* [12]. Since then, research on facial pits has advanced significantly, focusing on their structural, physiological, molecular and behavioural aspects [13–20]. These specialized organs appear as loreal depressions (between the eye and nostril) in pit vipers (Crotalinae) and as multiple labial slits in most pythons (Pythonidae) and some boas (Boidae) [21–23]. Though functionally similar, loreal pits are more structurally complex and at least four times more sensitive than labial pits [24,25], possibly explaining why pythons and boas have multiple pit pairs, while pit vipers have only one. Both types aid in prey detection and thermoregulation, allowing snakes to sense sudden thermal changes and seek appropriate refuges [26–28]. While true vipers (Viperinae) and non-pit-bearing boas and pythons can also sense heat [24,29], pit-bearing species are particularly adept at detecting thermal radiation [18,30].

Given the numerous advantages of loreal pits and their potential role in ecological specialization, they have been proposed as a key innovation in these groups of snakes, which might have affected the diversification rates [31–33]. The concept of what defines a key innovation has been fluid [34,35]. Originally developed to describe traits that facilitate the radiation of a clade into new adaptive zones, it has since been expanded to encompass evolutionary changes that lead to increased diversification within the clade [31]. Thus, if the emergence of pits indeed allowed these snakes to exploit novel ecological niches, it might have left macroevolutionary signatures such as shifts in speciation or net diversification rates.

Previous studies have investigated the role of environmental and ecological factors in shaping the evolution of heat-sensing pits, especially emphasizing the role of prey type, hunting mode and habitat thermal properties [36,37]. Canopies, for instance, experience rapid heating due to direct sunlight during the day and significant cooling due to wind and radiative heat loss at night [38,39]. This makes arboreal microhabitats more thermodynamically labile, with wider temperature fluctuations that could challenge organisms to maintain thermal balance while foraging or seeking refuge [39]. On the other hand, terrestrial habitats tend to offer more homeostatic and predictable thermal conditions [38,40]. Given this thermal complexity in arboreal habitats, it is plausible that the presence of highly sensitive and specialized pit organs might allow snakes to detect subtle temperature variations in their environment, aiding in prey detection and the identification of thermally favourable refuges within the dynamic canopy. Schraft *et al.* [28] demonstrated that ambush hunting enables pit-bearing snakes to maximize their IR-sensing capabilities by positioning themselves in environments with strong thermal contrasts, such as near warmer shrubs or areas with distinct temperature transitions. This strategy enhances their ability to detect warm-blooded prey while minimizing energy expenditure by remaining stationary until prey is within striking distance. Snakes with pit organs utilize both visual and thermal cues for prey detection [41], with visual cues aiding in capturing a wide range of prey. However, the thermal sensitivity of the pits likely drives a preference for warm-blooded prey in nocturnal snakes, where visual cues are limited [28,42].

However, these observations are primarily based on studies of pit vipers, with little attention given to other pit-bearing lineages. Furthermore, while these ecological associations are hypothesized, they have not been systematically tested within a phylogenetic framework. In this study, we address these gaps by systematically exploring the relationship between the evolution of IR-sensing pits and ecological niches using a robust hypothesis-testing framework in a phylogenetic context. By integrating variables such as diet, habitat preference and hunting strategy of pit-bearing non-crotaline snakes (pythons and boas), we offer a broader evolutionary perspective on this unique trait.

Pythonoidea and Booidea are among the most species-rich (~104 extant species) snake superfamilies [43,44], with Pythonoidea distributed in the Old World and Booidea globally. These groups are phenotypically and ecologically diverse [44] and show significant size variation—from the 70 cm *Antaresia perthensis* to the 32 feet *Malayopython reticulatus* [45,46]. Members of some Booidea genera, such as *Eryx*, *Candoia* and *Exiliboa*, exhibit dwarfism [47]. Their ecological diversity spans biomes such as tropical forests to deserts and scrublands [47]; microhabitats such as terrestrial, arboreal and fossorial [44,47] and diet preferences that include both ectothermic and endothermic prey [44,47,48]. This ecological heterogeneity and wide distribution, coupled with selective evolution of pits across lineages, offers opportunities to explore the ecological correlates and evolutionary consequences of heat-sensing pits.

We aim to investigate the following questions:

(1) What are the ecological contexts in which pits confer an advantage in boas and pythons? Specifically:
  (a) Is the evolution of pits linked to the evolution of specific dietary habits? We hypothesize that the evolution of pits might be associated with a preference for endothermic prey.
  (b) How do pits influence habitat preference? We hypothesize that the emergence of pits might be linked to the evolution of arboreal habitat use.
  (c) What is the effect of pits on hunting strategy? We hypothesize that the evolution of pits could have driven a preference for ambush hunting.
(2) Is the emergence of IR-sensing pits associated with increased species diversification in boas and pythons?

## 2. Material and methods

### (a) Phylogeny of boas and pythons

We obtained the maximum clade credibility (MCC) tree from a recent study [43], which represents the most updated phylogeny of squamates. Title *et al.* [43] first generated a genomic backbone using 1018 selected species and then incorporated additional species using legacy markers, resulting in a comprehensive phylogeny of squamates with approximately 65% coverage. We downloaded this MCC chronogram and pruned other groups to retain only the Pythonoidea and Booidea superfamilies.

This pruned chronogram, comprising 92 tips (57 Booidea and 35 Pythonoidea species), represents the most comprehensive taxon coverage available for these groups to date, covering approximately 90% of their known diversity. Branch support details for this tree are provided in Title *et al.* [43]. The final pruned chronogram was used for all subsequent analyses.

### (b) Data collection

We undertook comprehensive data mining to compile trait data for chosen species from SquamBase [49], the Reptile Database (www.reptile-database.org), various pet trade websites and published literature. For each species, we documented the following traits: presence or absence of pits, hunting behaviour (ambush and active hunting), habitat preference (arboreal, terrestrial and fossorial), activity patterns (diurnal and nocturnal), dietary habits (ectothermic and endothermic diet), reproductive mode (viviparous, oviparous and ovoviviparous), average snout–vent length (SVL) and primary biome of occurrence. The complete dataset for all species, along with the sources of information, is made available in the electronic supplementary material, §S1.

Certain traits in pythons and boas resist straightforward classification. For example, *Chilabothrus angulifer* employs both ambush and active hunting strategies [50] (see electronic supplementary material, §S1). Similarly, dietary preferences in snakes span a wide spectrum, encompassing both endothermic and ectothermic prey. In the case of pythons, we categorized each species’ diet as either ectothermic or endothermic based on the proportion of warm-blooded prey consumed, using a 50% threshold as a cutoff [48]. For boas, dietary classification was based on the predominant prey types documented in the literature.

Habitat classification presented similar challenges. While many species exhibit strict habitat preferences, several python and boa species do not conform neatly to categories such as arboreal, terrestrial or fossorial. For example, species classified as ‘semi-fossorial’ or ‘semi-arboreal’ in SquamBase required additional interpretation. In these cases, we referred to electronic supplementary literature and natural history observations to identify the habitat classes these snakes predominantly occupy. We categorized semi-fossorial snakes as terrestrial+fossorial, and semi-arboreal snakes as terrestrial+arboreal in cases where their habitat use showed no clear preference for one over the other. Consistent with our approach to diet coding, snakes predominantly associated with a specific habitat type were coded as occupying that habitat.

To account for the inherent subjectivity in these classifications, we adopted two coding schemes during data processing: a strict coding scheme and a relaxed one. Results presented in the main text are based on the strict coding scheme, while those derived from the relaxed coding are provided in the supplementary materials to ensure robustness and transparency against potential biases.

Under strict coding, the following conventions were applied: For hunting behaviour, species primarily using ambush hunting were coded as state 1, whereas those employing active hunting or a combination of both were coded as state 0. Habitat preference was coded as state 1 for species that were strictly arboreal, and state 0 for those with other or mixed habitats (arboreal, terrestrial, fossorial or aquatic). In terms of diet, species that predominantly fed on endotherms were coded as state 1, while those with an ectothermic bias or mixed diets were coded as state 0. Finally, strictly nocturnal species were coded as state 1, and all other species were coded as state 0.

Under relaxed coding, for hunting behaviour, species exhibiting any presence of ambush hunting (including combinations with active hunting) were coded as state 1, while other modes were coded as state 0. Arboreality was coded as state 1 when present and state 0 when absent. Diet was coded as state 1 if endotherms were included in the diet, and state 0 otherwise. Additionally, nocturnal activity was coded as 1 if present, including cases where it co-occurred with diurnal or cathemeral patterns, while taxa lacking nocturnal activity were coded as 0.

A limitation of the strict and relaxed coding schemes lies in the *a priori* selection of a focal trait state based on our hypotheses. For instance, we designate ambush hunting as the focal state for hunting mode, endothermic diet for diet and nocturnality for diel activity. This approach allows us to structure our analyses around specific hypotheses but narrows the scope, potentially limiting our ability to detect associations with other trait states. This issue is particularly pronounced for multi-state traits such as habitat, which includes categories like arboreal, fossorial, terrestrial and aquatic. For example, when encoding habitat, ‘arboreality’ is defined as state 1, while ‘terrestriality', ‘fossoriality' and ‘aquatic’ are grouped together as state 0. Consequently, if other habitat states influence the presence or absence of labial pits, our coding scheme may fail to capture those effects.

To address this concern, we conducted additional analyses using strict and relaxed coding schemes separately for fossorial and terrestrial habitats. This allowed us to test their specific associations with labial pits and mitigate potential biases in habitat-related traits. However, this was not done for aquatic species because there are very few aquatic species (just five) in the dataset.

However, this issue does not arise for binary traits such as hunting mode, diet or diel activity. In these cases, the strict coding for one trait state (e.g. ambush hunting) inherently serves as the relaxed coding for the alternative state (e.g. active hunting), and vice versa. Therefore, if the non-focal state has a significant effect, it will still be captured by the complementary coding scheme.

Our final dataset (available in electronic supplementary material, §S1) included ecological and trait data for 91 taxa, representing approximately 88% of extant species from the superfamilies Booidea and Pythonoidea. The strict and relaxed coding datasets are available from electronic supplementary material, §S2.

**Figure 1.**
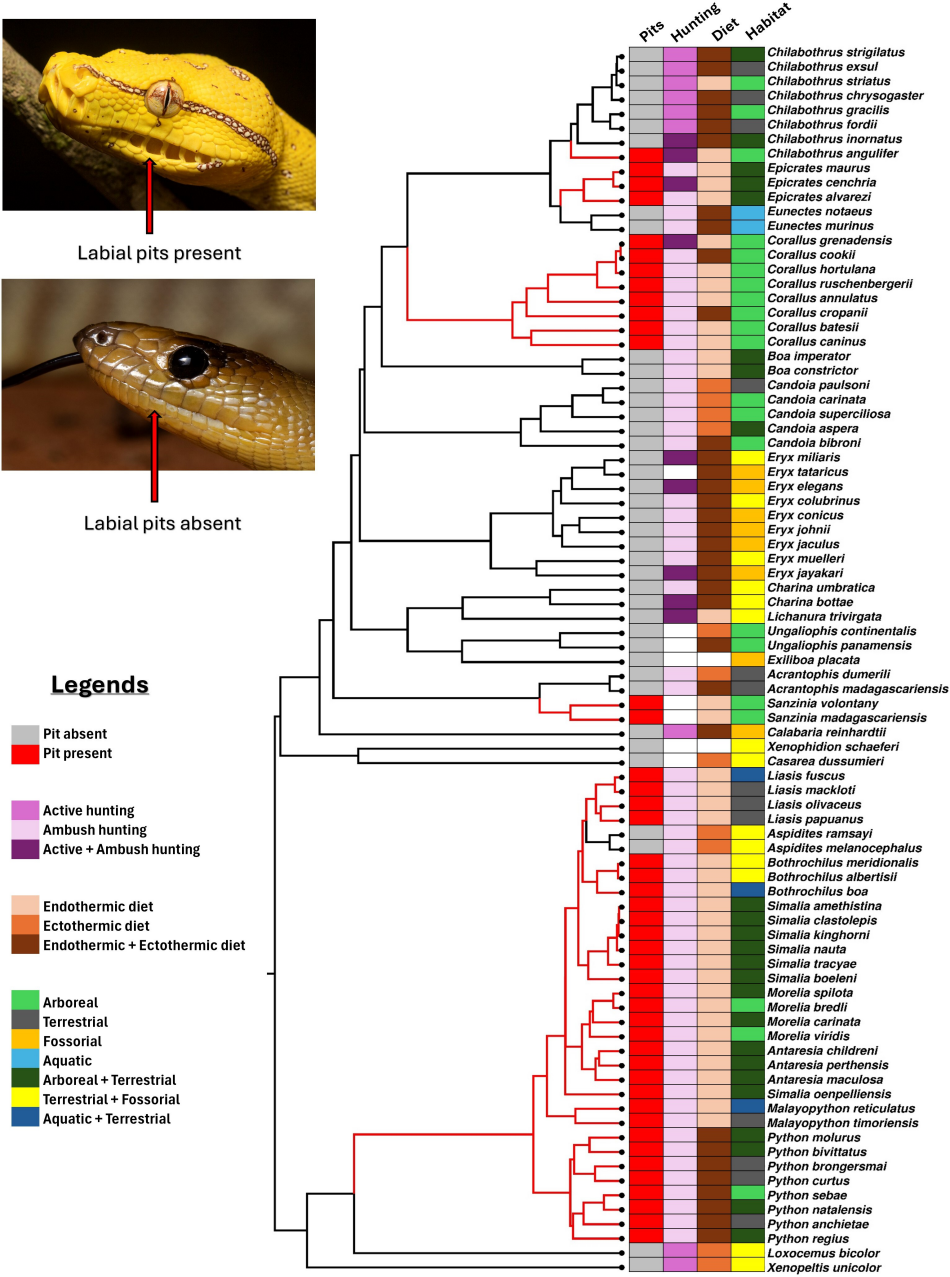
Trait states (labial pits, hunting mode, diet and habitat) mapped at the tip of the phylogeny. Branches along which labial pits evolved are highlighted in red. White states indicate lack of data. Photographs by Damien Esquerre.

The distribution of some of the trait sates across the phylogeny of boas and pythons is shown in figure 1.

### (c) Ancestral state reconstruction

To explore the evolution of labial pits, we performed ancestral state reconstruction using the phytools package [51]. Each taxon was coded as either 0 (absence of pits) or 1 (presence of pits), for which we tested the ER (equal rate) and ARD (discrete rate) models. The likelihood ratio test suggested that ER model was a better fit to the data than ARD model. Subsequently, we generated 1000 stochastic character maps based on the ER model and summarized them employing the simmap function in phytools.

### (d) Testing correlations and causal models with fitPagel

We examined correlations between the presence of labial pits and selected ecological and biological traits using the fitPagel function from the phytools package. Two evolutionary models were compared: the independent model, which assumes no correlation between the two binary traits (involving only four transition rates), and the dependent model, which assumes correlated evolution of the traits (involving eight possible transition rates between the four character states of the two variables). Model selection was based on a likelihood ratio test and the Akaike information criterion (AIC). To assess the association between labial pits and SVL, we performed a phylogenetic ANOVA (pANOVA) using the phytools package.

Once we identified a significant correlation, we delved deeper to understand the nature of the relationship between the traits. Specifically, we aimed to determine whether one trait influenced the other, or if the traits were interdependent. To test this, we developed four models:

– *Null (independent) model*: Assumes that the gain and loss of both traits occur independently.
– *Dependent pits model*: Assumes that the gain and loss of labial pits depend on the transition rates of the other trait.
– *Dependent trait model*: Assumes that the gain and loss of the other trait depend on the transition rates of labial pits.
– *Interdependent model*: assumes that the gain and loss of both traits influence each other.

We used the fitDiscrete method in the fitPagel function to calculate the AIC values for each model. The best-fitting models were identified based on their Akaike weights.

Additionally, we assessed pairwise correlations among all predictor variables using the fitPagel function.

### (e) Phylogenetic path analysis

We further investigated the strength and directionality of causal relationships among three discrete variables correlated with each other: the presence of labial pits, arboreality and an endothermic diet. To account for non-independence due to shared ancestry, we conducted phylogenetic confirmatory path analysis using the d-separation method [52]. This approach also provided insights into the interaction coefficients between variables, considering their mutual effects [53].

The analysis was performed using the R package *phylopath* [54], which employs a phylogenetic logistic regression approach. We defined 12 models representing possible relationships among the three variables as directed acyclic graphs. A detailed explanation of all the models and the rationale behind them are provided in the electronic supplementary material, §S3 (figure S3 and section S3) . These models were ranked based on the C-statistics information criterion (CICc), and models with both ΔCICc ≤2 and *p* > 0.05 (means that the null model is not significantly better than these models) were averaged [53]. The best-fitting models were averaged and visualized with their standardized path coefficients and causal directions. Additionally, path coefficients and their confidence intervals were plotted for comparative analysis.

### (f) Diversification analysis

Trait-dependent diversification analysis was employed to investigate whether the presence of heat-sensing pits correlates with higher diversification rates. Trait-dependent diversification methods assess whether specific traits, such as heat-sensing pits, influence species diversification by examining their impact on speciation and extinction rates.

The analysis begins by estimating the probabilities of trait states in extant species and tracing them backwards through a phylogenetic tree. Parameters such as speciation, extinction and trait transition rates are used to calculate the likelihood of the observed trait distribution given the evolutionary history. This likelihood serves as a measure of how well the model explains the data. By optimizing these likelihoods, the parameter combination that best explains the data is identified, revealing the influence of traits on diversification dynamics [55].

We utilized hidden state speciation extinction (HiSSE) analysis through the R package *hisse* [56]. HiSSE incorporates hidden states to explore potential factors influencing diversification rates independently of the focal trait, allowing for the comparison of complex character-independent null models that modify diversification rates across clades. This approach also significantly reduces the type I error rate.

We evaluated 14 HiSSE models, accounting for sampling fraction (was set to 0.88). This set included two BiSSE-like HiSSE models, four null models with two and four hidden states, and eight character-dependent models with varying turnover rates and extinction fractions across two and four hidden states (electronic supplementary material, §S3 and table S6). Model selection was based on AIC scores to identify the best-fitting model.

Additionally, we employed missing state speciation and extinction (MiSSE) analysis [57] using the *hisse* package [56] as an alternative method to assess the association of diversification rates with the presence of pits. MiSSE is a trait-independent approach designed for estimating tip rates solely based on hidden states, providing a trait-free alternative for measuring rates across the phylogeny.

Given the size of the phylogeny, an exhaustive search among all possible MiSSE models (comprising 52 diversification parameters and 26 hidden states) was impractical. Instead, we limited our search to four hidden states and five estimated parameters, accounting for a global sampling fraction of 0.88. Using the MiSSEGreedy function, we evaluated models in chunks of two, starting with simpler models and progressing to more complex ones, based on AICc. The search terminated if no models within a chunk improved the delta AICc score difference (ΔAICc<2). Following the search, redundant models were pruned, and model-averaged tip rates were computed.

We extracted tip rates for all taxa and examined the association between tip rates and trait states (0 and 1) using PGLS ANOVA with the *phylolm* package [58].

### (g) Sensitivity analysis

To address uncertainty in age estimates and taxonomic relationships, we sampled 100 trees from the posterior distribution. We conducted fitPagel and phylogenetic path analyses on these trees using both strict and relaxed coding schemes. Results across the 100 trees were visualized to illustrate median values and parameter variability.

All analyses were carried out using the R statistical computing environment [59].

## 3. Results

### (a) Ancestral state reconstruction

Ancestral state reconstruction analysis indicated that the absence of heat-sensing pits was the ancestral condition figure 1. Heat-sensing pits evolved independently multiple times across various lineages. Within the superfamily Pythonoidea, labial pits originated once on the branch leading to the Pythonidae family, followed by their loss in the ancestral lineage of the genus *Aspidites*. In contrast, the superfamily Booidea exhibited no losses of heat-sensing pits, but multiple independent gains were observed. Specifically, pits evolved independently at least four times in the lineages leading to the genera *Sanzinia*, *Corallus* and *Epicrates*, and the species *Chilabothrus angulifer*. The simmap output showing the relative probability of trait states at every node can be found in the electronic supplementary material, §S3 and figure S1.

### (b) Correlation and causal model testing with fitPagel

The correlation analysis using the fitPagel function (table 1) on the strict coding dataset revealed strong associations between the presence of pits and both an arboreal habitat (likelihood ratio = 13.4, ΔAIC = 9.4, *p* = 0.00123) and an endothermic diet (likelihood ratio = 22.24, ΔAIC = 18.24, *p* < 0.001). However, no significant correlations were found between pit presence and other traits, including hunting mode, parity, diel activity, biomes and SVL. Similar results were obtained for the relaxed coding dataset (supplementary material, §S3 and table s1).

**Table 1.** Correlative tests as derived via Pagel’s test. *p*-values indicate that the presence of pits appears to have a strong correlation with the habitat preference and diet but not any other characters. Significant associations are highlighted in bold.

focal trait	other traits	likelihood ratio	*p*‐value	ΔAIC
heat-sensing pits	**habitat (arboreality**)	**13.4031**	**0.00123**	**9.4031**
	**diet**	**22.2389**	**<0.001**	**22.2389**
	diel activity	4.12	0.128	0.11846
	parity mode	1.3436	0.51	2.65638
	hunting mode	5.13	0.077	1.1295
	biomes	1.832	0.4	2.1679
	SVL	—	>0.1 (pANOVA)	—

Correlation testing using the strict and relaxed coding schemes with respect to terrestrial and fossorial habitat did not yield any significant correlation between these habitat types and presence of heat-sensing pits (electronic supplementary material, §S3 and table S2).

**Figure 2.**
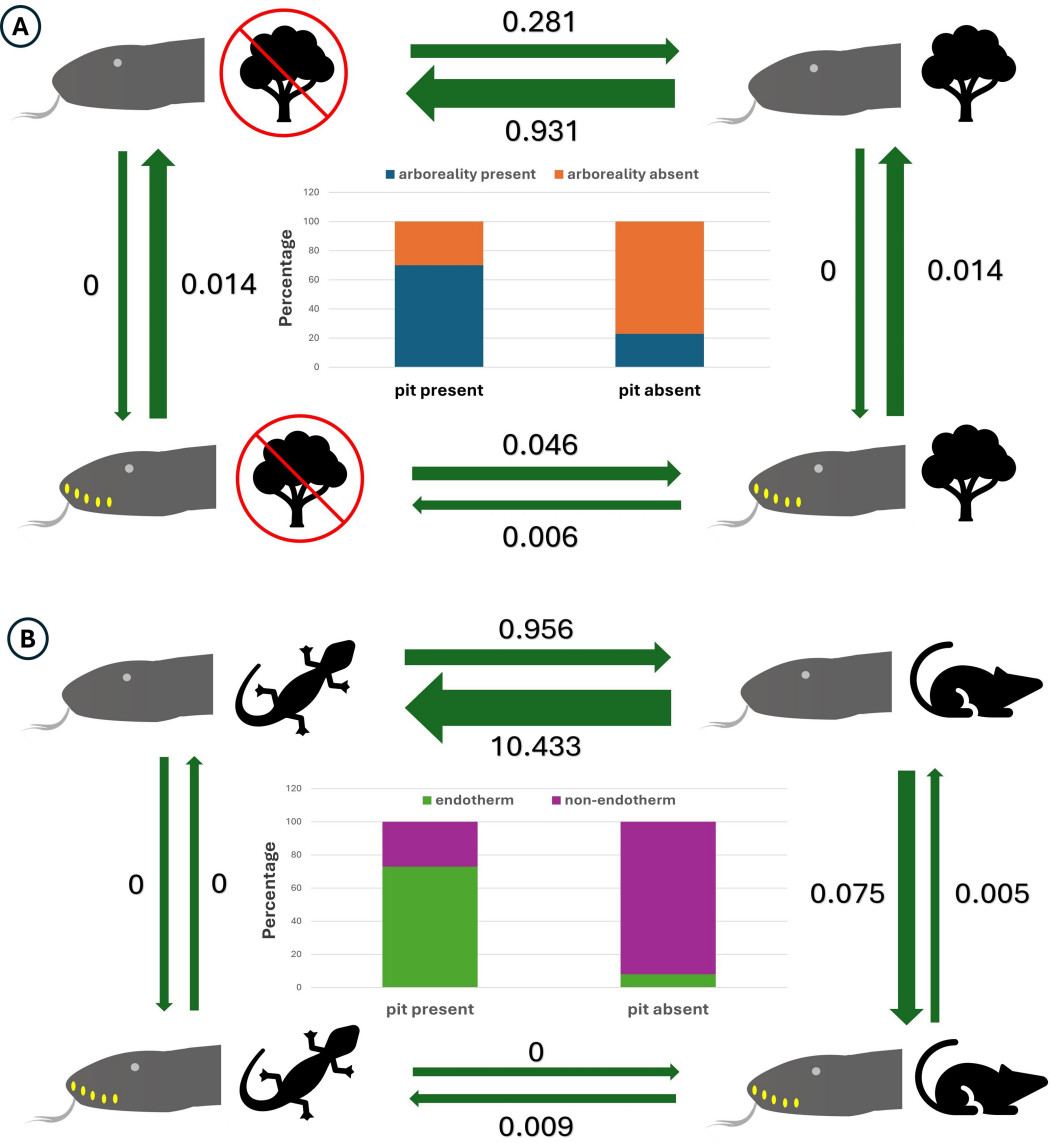
Predicted evolutionary transitions for the traits according to the best fitted model. Arrows are scaled according to the effect size. (A) Gain and loss of pits are unaffected by the presence or absence of arboreality. However, the rate of gain and loss of arboreality is different based on the presence or absence of pits. (B) Gain and loss of pits are only happening in the presence of an endotherm biased food habit. Also, the rate of shift from endothermic to ectothermic diet is much lower in the presence of pits.

We then explored the directionality of the significant relationships (table 2). For pits and arboreal habitat, the best-fitting model indicated that the evolution of habitat use was dependent on the presence of pits (AIC weight = 0.71). Transition rates estimated under this model (figure 2A) demonstrated that arboreality did not significantly affect the gain or loss of pits. Notably, the transition rate from an arboreal to a non-arboreal habitat was lower when labial pits were present, implying that pits may help preserve arboreal adaptations.

**Table 2.** Weighted AICs (relative probabilities) assess four different evolutionary scenarios for lifestyle and diet, where models of different evolutionary relationships are tested in each case. A scenario where habitat preference depends on pits is supported, whereas, in the case of diet, a model where the evolution of the pit and diet are interdependent appears as the best-supported model though marginally better than the model where only diet is dependent on pits. The best-supported models are highlighted in bold.

focal trait	other traits	dependency	Akaike weights (AICw)
heat-sensing pits	habitat (arboreality)	independent	0.0001939
		**habitat depends on pits**	**0.7121174**
		pits depend on habitat	0.1005947
		interdependent	0.187094
	diet	independent	0
		diet depends on pits	0.4723245
		pits depend on diet	0.0206048
		**interdependent**	**0.5070707**

Regarding pits and diet, the highest Akaike weight (0.507) was assigned to an interdependent model where both traits evolved in tandem (table 2) . A model positing that only diet depended on pits was marginally less favoured (AIC weight = 0.472). Transition rates illustrated in figure 2B indicated that gains and losses of pits were predominantly linked to the presence of an endothermic diet, with a significantly lower transition rate from endothermic to ectothermic diets when pits were present.

These results pertain to the strict coding scheme, while similar strong correlations were observed in the relaxed coding scheme (electronic supplementary material, §S3 and table S3). The fitPagel analysis suggested that pits influenced habitat, whereas the model in which diet influenced the evolution of pits was the best fit for dietary interactions (electronic supplementary material, §S3 and table S3). The transition rates under the relaxed coding schemes are presented in electronic supplementary material, §S3 and figure S2.

Further correlation analysis among predictor variables identified significant associations between habitat and diet, as well as between habitat and biomes (electronic supplementary material, §S4). To quantitatively evaluate the extent and directionality of interactions among pits, diet and habitat, we conducted a phylogenetic path analysis, excluding biomes due to their lack of correlation with labial pits.

### (c) Path analysis

Phylogenetic path analysis revealed several positive interactions among the examined variables. Model 11, which posited that both the presence of pits and habitat influenced diet, emerged as the best-fitting model (electronic supplementary material, §S3, table S4 and figure s4). However, Models 3 and 4—where diet influenced pits, and pits influenced habitat or both diet and habitat, respectively—also met the ΔCICc≤2 threshold. However, since these two models had the same likelihood score, we retained only one (model Three) to avoid redundancy. Figure S4 (electronic supplementary material, §S3) illustrates the model weights alongside *p*-values, with bars indicating significant results (significance denoting rejection). The results of the model comparison for the strict coding scheme can be found in the electronic supplementary material, §S3 and table S4.

**Figure 3.**
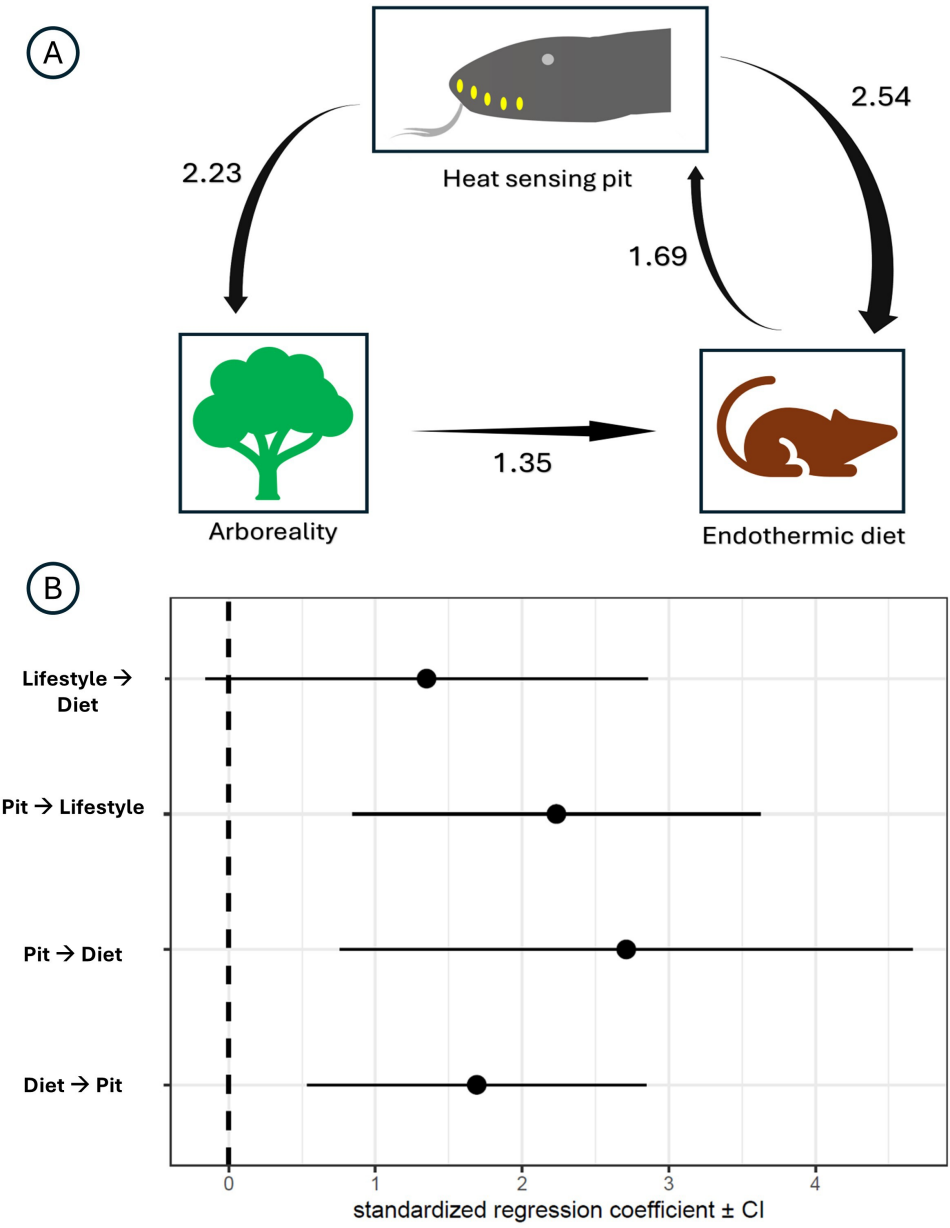
(A) Interaction coefficients from model averaging. Arrows are scaled according to the effect size. (B) Standardized values of the coefficients along with the confidence intervals.

These top models (ΔCICc≤2) were averaged, with the standardized interaction coefficients among the variables presented in figure 3A, Notably, the analysis suggested that the evolution of an arboreal habitat and endothermic diet was primarily driven by the presence of labial pits, rather than the reverse. Specifically, the strongest interaction was the influence of pits on dietary evolution, followed by their impact on arboreal adaptations. The impact of diet on pit ranked third, while the effect of habitat on diet was the weakest, indicating that diet was influenced by habitat. The coefficients for these interactions, along with their estimated confidence intervals, are detailed in figure 3B.

Interestingly, the path analysis results for the relaxed coding scheme mirrored those of the strict coding scheme (electronic supplementary material, §S3, table S5 , figures S5 and S6), except for one critical distinction regarding the habitat–diet interaction. Under the strict coding scheme, habitat exhibited a positive effect on dietary evolution (arboreality associated with endothermic diet), whereas the relaxed coding scheme indicated a negative effect (arboreality associated with ectothermic diet) (electronic supplementary material, §S3 and figure S6).

### (d) Diversification analysis

The HiSSE analysis indicated weak support for a trait-dependent diversification scenario (electronic supplementary material, §S3 and table S6). The best-fitting model was a trait-independent model with two hidden states (Model 3, electronic supplementary material, §S3 and table S6). This finding suggested that the observed rate heterogeneity across the phylogeny was not influenced by the presence or absence of labial pits but rather due to some unobserved or hidden state. The second-best model was a trait-dependent model with two hidden states (Model 10, electronic supplementary material, §S3 and table S6), which proposed that both the observed state (presence or absence of pits) and the hidden states (labelled A and B) had different diversification rates. Although Model 3 was not significantly better than Model 10 (ΔAICc = 0.79), the null model had fewer parameters, favouring a simpler explanation. Thus, while the HiSSE analysis suggested the existence of diversification rate differences across the phylogeny, these could not be attributed to labial pits alone and were likely driven by other factors.

**Figure 4.**
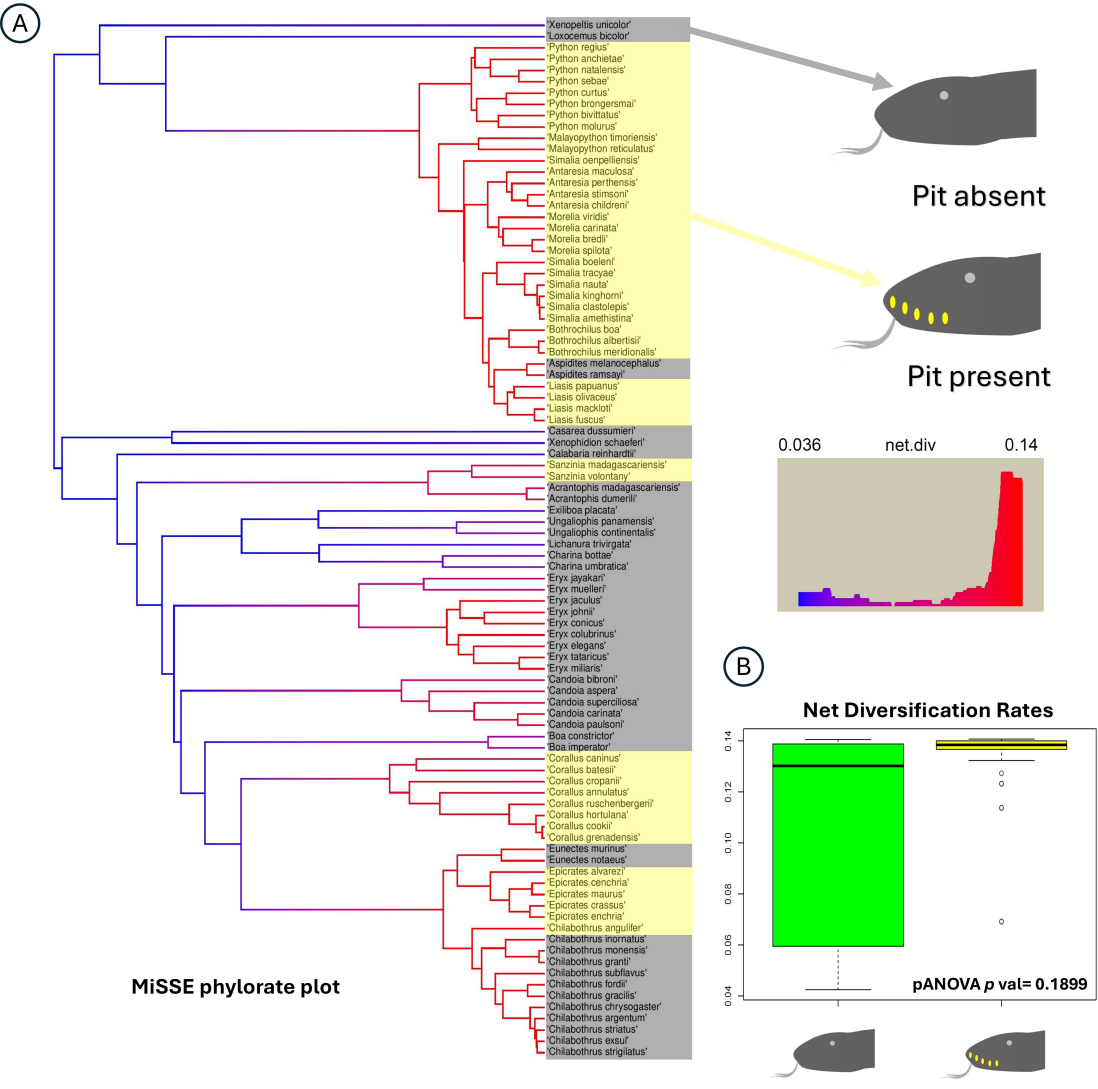
MiSSE phylorate plot showing lineage wise net diversification rates in figure 4A. Figure 4B is the box-plot representing the distribution of the net diversification rates for the presence and absence of heat sensing pits.

To further test for trait-dependent diversification using a different method, we employed MiSSE analysis. The trait states for both traits were plotted at the tips, alongside the phylorate plot estimated by MiSSE (figure 4A). PGLS ANOVA on the estimated tip rates did not reveal a significant association between the presence of pits and diversification rates. The box plot (figure 4B) showed generally higher net diversification rates in taxa with pits (~0.14), while rates for taxa without pits varied widely, ranging from 0.14 to 0.05. However, this observed association was not statistically significant (*p* = 0.1899).

### (e) Sensitivity analysis

The fitPagel analysis, conducted across 100 trees for the strict coding dataset, consistently supported a model where habitat use patterns depended on the presence of pits, providing strong evidence for this relationship (electronic supplementary material, §S3 and figure S7). Additionally, the analysis favoured a model in which the evolution of diet was contingent on the evolution of pits, with robust support across all 100 trees (electronic supplementary material, §S3 and figure S8). For the relaxed coding scheme, results aligned closely with the strict coding findings; however, the relaxed coding favoured a model suggesting that the evolution of pits was influenced by diet, showing high support (electronic supplementary material, §S3 and figures S10 and S11).

Phylogenetic path analysis was also performed across 100 trees, visualizing interaction coefficients as box plots (electronic supplementary material, §S3 and figure S9). Coefficient values displayed minimal variation across runs. The strongest interaction identified was of pits on diet (average = 3.32 ± 0.025), followed by the influence of diet on pits (average = 1.99 ± 0.015) and the effect of pits on habitat (average = 1.72 ± 0.014). The weakest interaction was of habitat on diet (average = 0.66 ± 0.03), with all interactions remaining positive.

For the relaxed coding dataset, results diverged slightly from the strict coding (electronic supplementary material, §S3 and figure S12). The effect of diet on pits remained the most substantial interaction (average = 4.31 ± 0.03), followed by pits influencing diet (average = 2.21 ± 0.18) and pits affecting habitat (average = 1.87 ± 0.07). The weakest interaction was of diet on habitat (average = 0.87 ± 0.06), with all interactions positive. Notably, a negative interaction was observed of habitat on diet (average = −1 ± 0.007) in the relaxed coding scheme.

All the data and scripts used for the analyses are available from electronic supplementary material, §S5.

## 4. Discussion

Despite several experimental and molecular studies on heat-sensing pits in boas and pythons, their evolutionary implications have so far received little attention. Our phylogenetic comparative analyses based on a large and robust dataset shed light on the effect of these IR-sensing pits on the macroevolutionary implications and the ecological attributes influencing their evolution.

### (a) The multiple evolution of labial pits

Our findings indicate that heat-sensing labial pits evolved independently at least five times within the Pythonoidea and Booidea superfamilies. The molecular basis for IR sensing in snakes has been associated with the transient receptor potential ankyrin 1 (TRPA1) gene, a heat-sensitive calcium ion channel critical for the thermal physiology of vertebrates [60–62]. However, the thermal activation threshold of TRPA1 varies among snakes: in IR-sensing species like crotalines, pythons and boas, TRPA1 activates at lower temperatures (~28°C for crotalines and~30°C for pythons and boas), while in non-pit snakes, it activates at higher temperatures (~37°C), rendering it less sensitive.

Structural studies have identified amino acid substitutions at three sites in TRPA1 of pit-bearing snakes [60,61]. These sites are highly conserved across vertebrates but have undergone positive selection in lineages with IR-sensing pits [60]. This evidence suggests an association between these molecular changes and common ecological factors, which may contribute to potential advantages.

Our ancestral state reconstruction analysis supports a scenario involving five gains and one loss (figure 1). However, an alternative scenario with three gains and three losses—specifically in *Aspidites*, *Eunectes* and *Chilabothrus* (excluding *C. angulifer*)—is equally parsimonious. Despite this equivalence in parsimony, the biological plausibility of the five-gain and one-loss scenario aligns more closely with the complexity of the trait under investigation. Labial pits are anatomically intricate structures comprising specialized trigeminal nerve fibres and blood capillaries, as noted by several studies [60]. Developing such a sophisticated feature likely requires substantial evolutionary innovation, while its loss would entail significant reorganization of the nervous and circulatory systems in the labial region. These constraints suggest that trait losses are likely to be less frequent.

### (b) Labial pits, endothermic diet and arboreality

The environment surrounding an animal emits two primary types of photons: reflected photons, which fall within the ultraviolet and visible spectra; and emitted photons, predominantly in the IR range [17]. Labial pits, a distinctive feature of some booid and pythonid snakes, grant them the remarkable ability to detect IR radiation, providing a significant advantage over animals reliant solely on visible cues for prey detection.

Infrared-sensing receptors are also present in booid species like *Boa constrictor* and *Eunectes murinus*, which lack labial pits [23]. However, pit organs exhibit a more extensive nerve supply, a greater number of receptors, a denser capillary network and a thinner epidermis compared to surrounding tissues, enhancing their sensitivity and effectiveness [11,21].

Fossil evidence, such as *Eoconstrictor fischeri* from the Messel region, suggests that labial pits may have evolved early in booid snakes [63]. Analysis of these primitive pit-bearing fossils indicates that they were predominantly terrestrial, had larger body sizes and consumed ectothermic prey, whereas smaller fossilized boas lacked pit organs [63]. This pattern suggests a potential correlation between body size, habitat use and diet with the evolution of pit organs in booid snakes. Specifically, some of these primitive pit-bearing fossils were significantly larger than their non-pit-bearing counterparts, with an ectothermic diet, and a primarily terrestrial lifestyle.

Our findings, however, reveal a contrasting pattern in modern pit-bearing snakes, showing a strong association between labial pits, arboreal habitat use and an endotherm-biased diet. This suggests that while early pit-bearing snakes did not exhibit a significant link between pits, arboreality and endothermic prey, the selective advantages conferred by IR sensing may have driven a gradual ecological shift over time. As a result, lineages with labial pits likely transitioned towards arboreal habitats and an endotherm-biased diet, where thermal contrast would have enhanced foraging efficiency as well as thermoregulation.

Recent behavioural studies support this, demonstrating that snakes with labial pits excel at detecting endothermic prey against cooler, arboreal backgrounds [37]. These findings imply that although the initial evolution of IR-sensing pits may not have been directly tied to ecological factors like diet or habitat, their presence became increasingly advantageous in certain environments. Consequently, selection likely favoured lineages that occupied arboreal niches and hunted endothermic prey, reinforcing the ecological shift observed in modern species.

Our data further support the idea that the presence of pits influences habitat choice and dietary preferences rather than the reverse. While diet plays a role in the gain and loss of labial pits, our results suggest that pits exert a stronger influence on dietary and habitat transitions, potentially explaining the divergence between fossil and extant pit-bearing snakes.

Future research could benefit from exploring the detailed patterns of pit evolution, particularly the variations in the number and positioning of pits. For example, *Morelia viridis* possesses pits both in the upper and lower jaw, while *Antaresia childreni* and several other taxa have them exclusively in the lower jaw [63]. The biological implications of these variations remain unexplored.

### (c) Diversification rates and pits

Diversification rate measurements in vipers indicate an increase in speciation rate among crotalines (pit vipers) compared to viperines (non-pit vipers). However, this shift in speciation cannot be attributed solely to the emergence of loreal pits; instead, it probably reflects a combined effect of their development alongside dispersal into new geographic regions [64]. Similarly, our results reveal significant diversification rate heterogeneity within the Booidea and Pythonoidea phylogeny (figure 4), yet these rate shifts are not correlated with the presence or absence of labial pits.

Such absence of higher diversification rates linked to heat-sensing pits suggests that the evolution of this complex trait likely did not drive increased speciation rates in snakes. This finding challenges our expectations, especially given the intricate histological structure of IR-sensing pits, which have predominantly only been gained, with rare losses in extant snakes, aside from *Aspidites*.

While we did not find a direct association between diversification rates and labial pits, it is inappropriate to conclude that pits lack influence on the macroevolution of these snakes. Labial pits have significantly shaped the ecology of these species, affecting their habitats and dietary preferences, which in turn impact their evolutionary trajectories.

### (d) Are labial pits a key innovation?

The concept of key innovation has evolved significantly since its introduction by Alden Miller in 1949 [34,35]. Initially defined as any novel trait, whether behavioural or physiological, that allowed taxa to exploit previously inaccessible ecological niches [65], the definition has since shifted to a more quantitative framework with the advancement of phylogenetic methods. Key innovation is now understood as a trait that directly enhances net diversification rates [31–33]. However, recent studies have pointed out the limitations of this definition, emphasizing that a transition to a novel ecological niche does not always correlate with increased diversification rates [66–68]. Consequently, there is a renewed emphasis on the classical, ecologically focused definition of key innovation [69].

The evolution of labial pits in boas and pythons exemplifies Miller’s original concept of key innovation. Our evolutionary model fitting indicates a significant association between the emergence of these pits, an endothermic diet and an arboreal habitat. The fitPagel and path analysis further support the idea that the evolution of labial pits facilitated a transition to a novel ecological spectrum characterized by arboreality and a bias toward endothermic prey. Thus, based on these findings and the ecological definition of key innovation, we argue that the IR-sensing labial pits in boas and pythons indeed represent a case of key innovation.

In contrast, reaching the same conclusion for loreal pits in pit vipers (crotalines) is more complex. Given that loreal pits evolved only once in crotalines, applying a similar analysis is challenging. Ancestral niche reconstruction could provide valuable insights into this question. Specifically, investigating shifts in climatic or ecological niches during the evolution of loreal pits would be particularly intriguing in this context.

### (e) Robustness and limitations

We acknowledge that our analyses reveal correlations among traits rather than causation [70]. Additionally, there are limitations related to trait coding in our study. As noted in the electronic supplementary material, , §S1, ecological characteristics such as diet, diel activity and habitat exist on a spectrum for many taxa, making binarization challenging. Moreover, fine-scale data is lacking for many taxa, which limits our ability to use continuous variables like habitat use, activity patterns or the proportion of endothermic versus ectothermic prey in the diet. We addressed this issue by employing both strict and relaxed coding schemes. The consistency of associations between labial pits, diet and habitat preference, across 100 randomly sampled trees using these two coding schemes, reinforces our confidence in the results.

## Data Availability

All the data and scripts used in this article are available from the supplementary materials [71].
